# Influence of 3D Structural Design on the Electrochemical Performance of Aluminum Metal as Negative Electrode for Li‐Ion Batteries

**DOI:** 10.1002/cphc.202400493

**Published:** 2024-09-30

**Authors:** Marco Ricci, Sergio Marras, Martin Krammer, Molaiyan Palanivel, Remo Proietti Zaccaria, Andrea Paolella

**Affiliations:** ^1^ Istituto Italiano di Tecnologia Via Morego 30 16163 Genova Italia; ^2^ Austrian Institute of Technology Giefinggasse 4 Vienna Austria; ^3^ Research Unit of Sustainable Chemistry University of Oulu FI-90570 Oulu Finland; ^4^ Università degli Studi di Modena e Reggio Emilia Dipartimento di Scienze Chimiche e Geologiche Via Campi 103 16163 Modena Italia

**Keywords:** Aluminium, Alloy, Magnesium, Anode, Battery, Lithium, 3D structure

## Abstract

Aluminum (Al) is one of the most promising active materials for producing next‐generation negative electrodes for lithium (Li)‐ion batteries. It features low density, high specific capacity, and low working potential, making it ideal for producing energy‐dense cells. However, this material loses its electrochemical activity within 100 cycles, making it practically unusable. Several claims in the literature support the idea that a dual degradation mechanism is at play. First, the slow diffusion of Li in the Al matrix causes the electrochemical reactions to be partly irreversible, making the initial capacity of the cell drop. Second, the stress caused by cycling make the active material pulverize and lose activity. Recent work shows that shortening the diffusion path of Li by 3D structuring is an effective way to mitigate the first capacity loss mechanism, while alloying Al with other elements effectively mitigates the second one. In this work, we demonstrate that the benefits of 3D structuring and alloying are cumulative and that a mesh made of an Al‐magnesium alloy performs better than both a pure Al foil and a foil of an Al−Mg alloy.

## Graphical abstract

The cycling stability of aluminum (Al) negative electrodes can be greatly enhanced by concurrently alloying the base material with other elements and modifying its microscopic structure. In this work, this is proven by comparing the cycling behavior and the structural evolution of pure Al foil, a foil of 5754 Al‐magnesium (Mg) alloy, and a mesh of 5056 Al−Mg alloy.

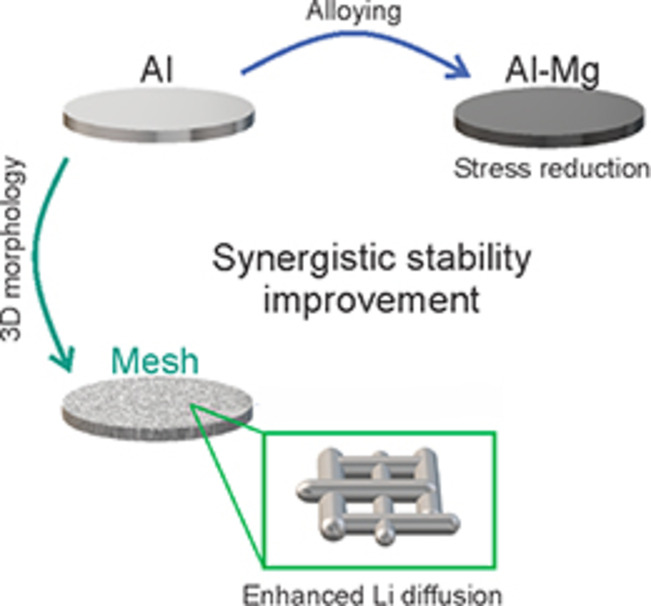

## Introduction

Our society needs safe and performant energy storage devices to assist with the replacement of fossil fuels.^[1]^ Lithium (Li) metal is regarded as the most promising active material for producing negative electrodes for next‐generation rechargeable batteries.^[2]^ Indeed, depending on the chemistry of the positive electrode (high‐voltage NMC, sulfur, or oxygen),^[3]^ replacing graphite‐based negative electrodes with metallic Li would at least double the specific energy of lithium‐based batteries. However, Li is expensive, and its excess in the battery must be minimized as much as possible, for instance, by improving the efficiency of cycling.^[4]^ Moreover, Li can quickly form needle‐shaped dendrites during repeated cycling, especially at high current densities, causing dangerous short‐circuits.[^5^, ^6^, ^7^, ^8^] The use of Li is also held back by its high reactivity with liquid electrolytes[^9^, ^10^] which leads to constant active lithium losses.[^11^, ^12^] An attractive alternative to Li electrodes is represented by materials that can alloy with Li metal at low potentials, such as silicon,[^13^, ^14^, ^15^] tin,[^16^, ^17^, ^18^] and aluminum.^[19]^ Thanks to their impressive specific capacity, these materials are a valid alternative to graphite‐based negative electrodes. Moreover, since these materials incorporate Li into their structure, their use avoids the formation of dendrites.^[8]^ Among the metals that can alloy with Li, aluminum (Al) is one of the most enticing: it is the most abundant metal in the Earth's crust,^[20]^ and due to its low density of 2.7 g cm^−3^, it is widely employed to produce both structural and decorative elements for the aerospace, automotive, packaging, and construction sectors. Beyond its capabilities as a structural material, its properties as an alloy‐type negative electrode for lithium‐ion batteries (LIBs) are outstanding, with the first reports of this application being published in the 1970s.^[21]^ Indeed, at room temperature, Li can react with Al to form the β‐Li_x_Al_1_ (0.916≤x≤1.16) phase.^[22]^ The theoretical potential for this reaction is about 0.38 V vs Li/Li^+ [23]^. From the stoichiometry of the lithiated phase, it can be calculated that the specific capacity of Al is quite impressive, reaching up to 1152 mAh g^−1^.^[22]^ If the lithiation process is carried out at extremely low rates or if the diffusion of Li into Al is promoted by increasing the temperature, Li‐rich phases such as Li_3_Al_2_ and Li_9_Al_4_ can form.[^24^, ^25^] This way, the theoretical capacity obtainable from this material becomes even higher. Unfortunately, Al‐based electrodes have demonstrated poor cycling stability, mainly due to the mechanical strain caused by cycling.^[26]^ This strain causes the electrode to pulverize and increase in porosity, leading to the loss of electrochemical activity.^[22]^ Another possible cause for the loss of active Li when employing a negative electrode made of Al has been hypothesized by Oltean et al.^[19]^ They have studied the electrochemical behavior of electrodeposited Al nanorods through cyclic voltammetry and concluded that β‐LiAl can become trapped in the structure of the Al electrode due to diffusional limitations: in fact, the diffusion of Li into Al is three orders of magnitude more sluggish than its diffusion into β‐LiAl.^[27]^ A possible way to promote the stability of these electrodes has been suggested by Chen et al.,^[28]^ who demonstrated that the purity of the metal used to produce the electrodes influences the composition of the Solid Electrolyte Interface (SEI), the Coulombic Efficiency (CE) during early‐stage cycling, as well as the stability of the electrode. Wang et al.^[29]^ have prepared several Al‐metal alloys (metal=tin, zinc, gallium), increasing the cycle life of Al foil negative electrodes by a factor of 2 thanks to the formation of a fine microstructure. Liu et al.^[30]^ have proposed an electrode made of an Al_94.5_In_5.5_ alloy: the presence of indium (In) improved the reversibility of the β‐LiAl phase during cycling. The alloy was tested as the negative electrode in full‐cell configuration with an argyrodite Li_6_PS_5_Cl solid‐state electrolyte and a LiNi_0.6_Mn_0.2_Co_0.2_O_2_ (NMC622)‐based positive electrode (active loading of 16 mAh cm^−2^) exhibiting a capacity of 2.0 mAh cm^−2^ after 500 cycles at a current density of 2 mA cm^−2^. These results make it clear that tuning the composition of the electrode, for instance by using Al‐based alloys instead of pure Al, is a viable way to bring a better performance to reality. Complementarily to alloying, which serves the purpose of increasing the stability of the electrode, is 3‐dimensional (3D) structuring to shorten the diffusion path of Li and facilitate the reversibility of the reaction, consequently reducing active lithium losses during the initial cycles.^[31]^ In this work, we explore the combined effect of the presence of a 3D structure and alloy elements on the performance of Al‐based electrodes. To do this, we tested and compared the performance of cells equipped with negative electrodes made of a pure Al foil, a foil made of an aluminum‐magnesium (Al−Mg) alloy, and a mesh made of an Al−Mg alloy.

## Materials and Methods

Aluminum foil (named Alu1 in the following) was purchased from MTI Corporation (product number EQ‐bcaf‐15u–280), while both the foil (named Alu2), made of 5754 Al−Mg alloy, and the mesh (named Alu3), made of 5056 Al−Mg alloy, were purchased from Goodfellow Cambridge Limited (product numbers AL01‐FL‐000150 and AL00‐MS‐000122).

The crystal structure of the samples were characterized by X‐ray diffraction (XRD). XRD patterns were recorded on a Malvern‐PANalytical Empyrean 3rd generation X‐ray diffractometer equipped with a 2.5 kW Mo Kα ceramic X‐ray tube operating at 60 kV and 40 mA and a GaliPIX3D solid‐state pixel detector in 1D mode. The diffraction patterns were collected with a focusing geometry in transmission mode using a Mo focusing mirror and a reflection‐transmission spinning sample stage (rotation speed=2 RPs). Air‐stable samples were sealed between two layers of 6 μm‐thick Mylar^®^ foil, while air‐sensitive samples were sealed between two layers of 7 μm‐thick Kapton^®^ foil lined with vacuum grease. A rough estimation of the average size of the crystalline domains of the β‐LiAl phase was obtained using the Scherrer equation.

The morphological evolution of the samples (before vs after cycling) was characterized by scanning electron microscopy (SEM). SEM imaging was conducted using a FEI Helios Nanolab 650 DualBeam scanning electron microscope‐focused ion beam (SEM‐FIB) system. The micrographs were acquired at an accelerating potential difference of 5 kV.

The chemical composition of the samples was characterized using X‐ray fluorescence (XRF). Multi‐point measurements were performed using a Bruker M4 Tornado micro X‐ray fluorescence (μ‐XRF) spectrometer equipped with an air‐cooled Rh‐anode X‐ray tube operated at 50 kV and 199 μA. The polychromatic beam is focused using poly‐capillary optics to a spot size down to 25 μm for Mo Kα. The detection of fluorescence is performed by an energy‐dispersive silicon drift detector with a sensitive area of 30 mm^2^ and an energy resolution <145 eV for Mn Kα. The measurements were carried out on the sample directly placed on the instrument platform in a chamber at a pressure of 20 mbar. The reported values were obtained by averaging the results of five measurements on five different positions on the same sample.

To test their stability as negative electrodes for lithium‐ion cells, Alu1, Alu2, and Alu3 were punched into disks having a diameter of 15 mm and used to prepare CR2032‐format coin cells. The coin cells were equipped with lithium iron phosphate (LiFePO_4_)‐based positive electrodes from NEI Corporation (NANOMYTE^®^ BE‐60E, 14.27 mg cm^−2^) punched to disks having a diameter of 14 mm, resulting in cells with a capacity of 3.85 mAh. The electrodes were separated by a Celgard 2400 membrane imbibed with 100 μL LP40 electrolyte (1 M LiPF_6_ in EC:DEC (1 : 1 v:v)). The cells were cycled using a BCS‐805 potentiostat from Biologic with the following protocol: formation at C/20 (2 cycles) and cycling at C/5. The upper and lower cutoff voltages were of 3.8 V and 2.5 V, respectively.

The voltage profile of Alu1, Alu2, and Alu3 during the lithiation/delithiation process has been measured by galvanostatic cycling in lithium‐metal cells. Alu1, Alu2, and Alu3 were punched into disks having a diameter of 15 mm and used to prepare CR2032‐format coin cells. The coin cells were equipped with lithium‐metal negative electrodes having a diameter of 16 mm and a thickness of 0.6 mm. The electrodes were separated by a Whatman GF/A membrane imbibed with 100 μL LP40 electrolyte (1 M LiPF_6_ in EC:DEC (1 : 1 v : v)). The cells were cycled using a BCS‐805 potentiostat from Biologic with the following protocol: formation at C/20 (2 cycles) and cycling at C/5. To recreate the electrochemical conditions experienced by the Al electrodes in full cells, the C‐rate was calculated based on the capacity of the full cells described in previous paragraph. For the same reason, lithiation was limited to 3.85 mAh, while the upper cutoff voltage was of 1.5 V to allow for complete delithiation.

## Results and Discussion

**Figure 1:**
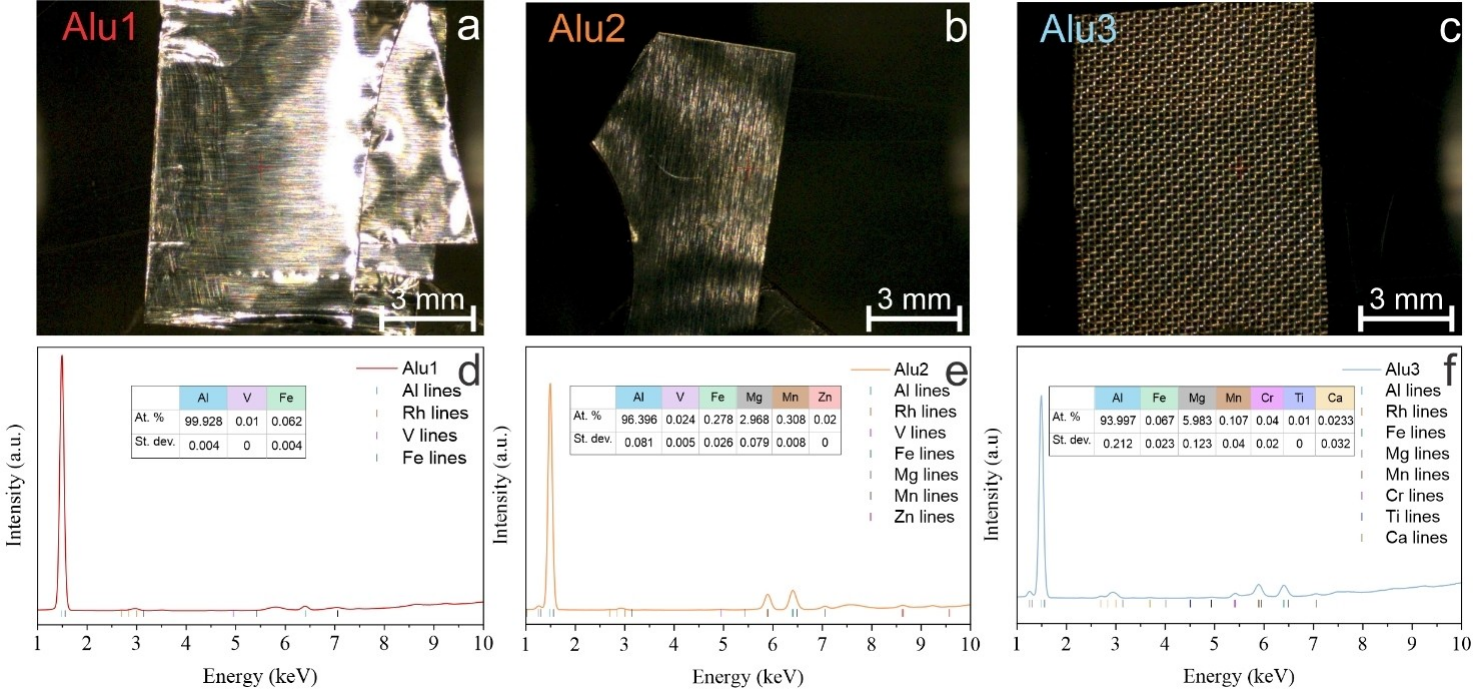
(a–c) Photographs of Alu1, Alu2, and Alu3 shot using the μ‐XRF spectrometer. (d–f) X‐ray fluorescence spectra and atomic composition (inset tables) of Alu1, Alu2, and Alu3.

Figure 1a–c shows photographs of Alu1, Alu2, and Alu3 shot using the XRF spectrometer. XRF was used to characterize the chemical composition of these samples. From its XRF spectrum (Figure 1d), Alu1 is calculated to be over 99.9 % pure Al with a small amount of vanadium (V, 0.01 %) and iron (Fe, 0.062 %) as impurities. Alu2, with its XRF spectrum shown in Figure 1e, shows a more varied composition than Alu1. This sample, having a nominal (vendor) composition of 97 % Al and 3 % Mg, actually contains 96.4 % Al and 2.97 % Mg. The impurities contained in the sample are transition metals like Fe, manganese (Mn), zinc (Zn), and V. Fe and Mn constitute 0.28 % and 0.31 % of the sample, respectively, while Zn and V are both present in the minute amount of 0.02 %. Alu3, with its XRF spectrum shown in Figure 1f, presents a higher amount of Mg than Alu2 (5.98 % vs 2.97 %), as expected from a 5056 alloy. Fe and Mn are present in minute amounts of 0.07 % and 0.11 %, respectively. Moreover, traces of Cr (0.04 %), Ca (0.02 %), and Ti (0.01 %) were detected. Overall, the chemical analysis by X‐ray fluorescence confirms that Alu1, Alu2, and Alu3 mainly differ in the relative amount of Mg, proving that these samples are suitable for assessing differences in the electrochemical performance arising due to the presence of this element.

**Figure 2:**
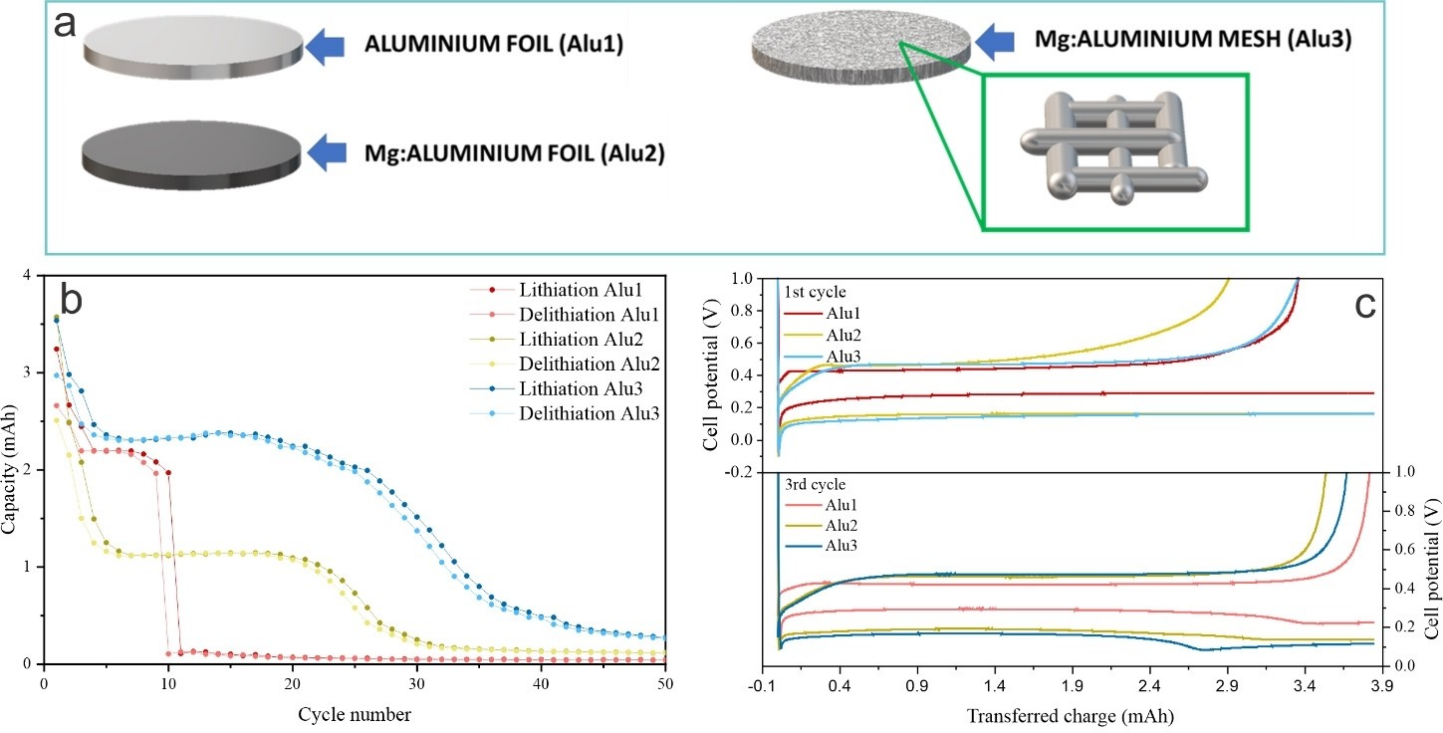
(a) Schematic representation of Alu1, Alu2, and Alu3. (b) Cycling stability of the samples in Alu//LFP full cells and (c) cell voltage profiles in lithium‐metal cells for selected cycles.

The stability of Alu1, Alu2, and Alu3 (Figure 2a) as negative electrodes for lithium‐ion cells was tested by galvanostatic cycling in the full‐cell configuration described in the Materials and Methods section. The results of the stability tests are shown in Figure 2b. The Alu1//LFP cell delivered a first discharge capacity of 2.66 mAh. Its capacity plateaued slightly above 2 mAh from the third cycle onwards and sharply dropped to 0 mAh during the tenth cycle. This result agrees with the results reported in the literature, showing that pure Al foil cannot withstand cycling with satisfying stability.^[19]^ The Alu2//LFP cell delivered a first discharge capacity of 2.5 mAh, comparably to the Alu1//LFP cell. However, the capacity of the Alu2//LFP cell dropped sharply over the initial cycles, plateauing at about 1.1 mAh from the sixth to the 18^th^ cycle. This capacity drop can be ascribed to the irreversible formation of LiAl, which, as described by Oltean et al.,^[19]^ becomes trapped in the Al electrode. In the case of Alu2 the trapping phenomenon is more severe than for Alu1 because the thickness of the former is about 13 times that of the latter, hindering the diffusion of Li out of the foil. The capacity further dropped from 1.1 mAh to a negligible value from the 18^th^ to the 30^th^ cycle. This means that the increased content of Mg could practically double the cycling stability of Alu2 compared to Alu1. The Alu3//LFP cell demonstrated a first discharge capacity of 2.97 mAh, slightly higher than the previous cells. In this case, the capacity drop during the initial cycles was milder than for the Alu2//LFP cell. The capacity plateau, less defined than in the previous cases, occurred between 2.3 and 2 mAh and lasted from the fourth to the 25^th^ cycle. The capacity dropped from the 25th cycle onwards, reaching a negligible value during the following ten cycles. The milder capacity drop during the initial cycles of Alu3 compared to Alu2 confirms the hypothesis of Crowley et al., who stated that shortening the diffusion path of Li can effectively prevent LiAl from becoming irreversibly trapped during the initial cycles,^[32]^ making structural failure the dominant capacity loss mechanism. Structural failure plays a significant role from the 25^th^ cycle onwards, leading to the observed capacity fading. In this regard, the high content of Mg in the alloy constituting Alu3 could delay the onset of structural failure, making this sample significantly more stable than the previous ones. In this regard, the difference between Alu1 and Alu3 is considerably more pronounced than between Alu2 and Alu3. This is because of the high purity of Alu1, which makes it the worst in terms of mechanical resilience. This confirms the claim of Li et al., who hypothesized that the addition of alloy elements to Al could mitigate the mechanical stress arising from the lithiation of this material by enhancing its diffusion toward the surface.^[33]^

The voltage profile of Alu1, Alu2, and Alu3 was measured by galvanostatic cycling in lithium‐metal cells as described in the Materials and Methods section. The objective was to understand how the morphology and composition of the Al electrodes affected their electrochemical behavior. The results are shown in Figure 2c for selected cycles (the first one at C/20 and the first one at C/5). It is noticeable that all the electrodes present a significant overpotential during lithiation. In fact, while the theoretical potential for this reaction is 0.38 V vs. Li/Li^+^, Alu1 lithiates at about 0.2 V vs. Li/Li^+^, while Alu2 and Alu3 do so at about 0.1 V vs. Li/Li^+^. On the other hand, the overpotential during delithiation is way less significant, showing that the growth of β‐LiAl is energetically unfavored with respect to the reverse process. Moreover, the overpotential for Alu1 is significantly less than for Alu2 and Alu3, probably due to its lower thickness which leads to a less pronounced ohmic polarization. When it comes to the nucleation of β‐LiAl, there is a clear difference between the three samples. In particular, Alu1 shows a nucleation overpotential for β‐LiAl of 0.35 V, while this value decreases to 0.25 V for Alu2 and 0.2 V for Alu3. It can be concluded that the presence of Mg in Al electrodes definitely facilitates the nucleation of β‐LiAl.

**Figure 3:**
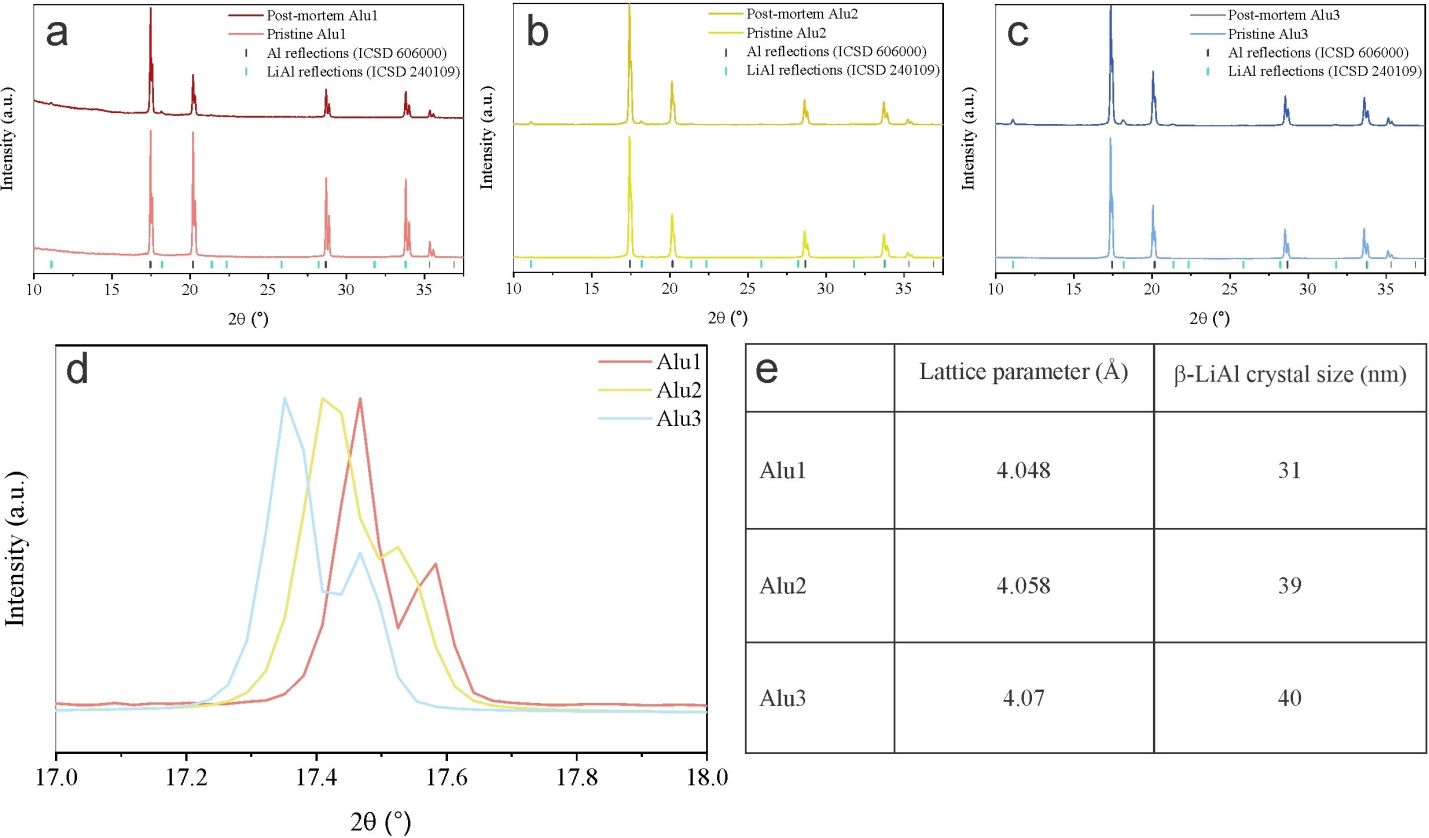
(a–c) X‐ray diffractograms of (a) Alu1, (b) Alu2, and (c) Alu3 before and after cycling. (d) Enlarged view of the highest intensity peaks of pristine Alu1, Alu2 and Alu3 showing how the peak progressively shifts to lower angles as the amount of Mg dissolved in Al increases. (e) Lattice parameters of Alu1, Alu2 and Alu3 calculated from the position of the diffraction peaks and size of the crystals of β‐LiAl calculated using the Scherrer equation.

**Figure 4:**
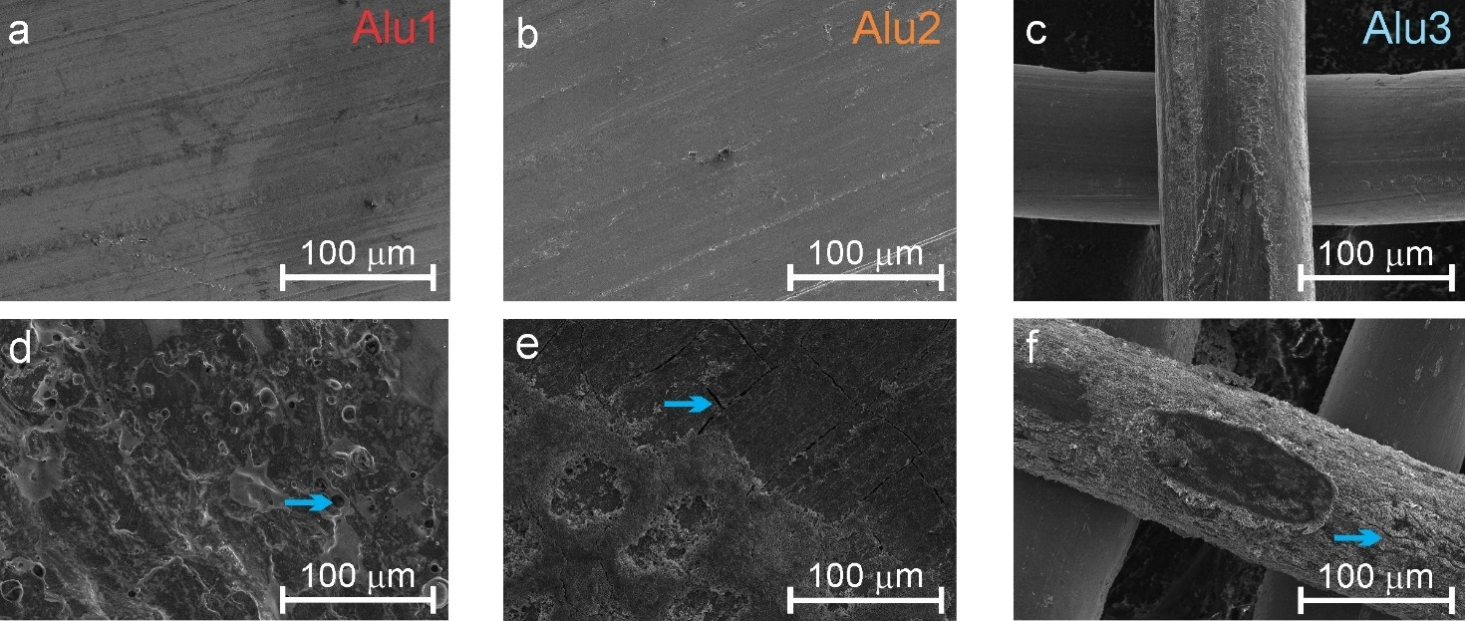
(a–f) Top‐view scanning electron micrographs of (a, d) Alu1, (b, e) Alu2, and (c, f) Alu3. (a–c) Micrographs of the pristine samples and (d–f) micrographs of samples retrieved after cycling.

The crystal structure of Alu1, Alu2, and Alu3 was analyzed before and after cycling by using XRD (results shown in Figure 3a–c): the pristine samples show the presence of a single phase related to pure Al (ICSD 606000), indicating that in samples Alu2 and Alu3, Mg is dissolved into the Al matrix. The absence of second phases, which could have a different electrochemical response than the matrix, makes the electrochemical behavior of the samples more readily comparable. As pointed out by Azon et al.^[34]^ the presence of Mg dissolved into Al significantly affects the lattice parameter of the cubic structure. In particular, as shown from the portion of the diffractogram of the pristine samples in Figure 3d, a higher concentration of Mg leads to a shift of the diffraction peaks to lower angles, meaning that the unit cell of the material is dilating. The lattice parameters calculated from the diffractograms of the pristine samples are reported in Figure 3e. After cycling, XRD revealed the presence of a second phase in the samples, related to the irreversible formation of β‐LiAl (ICSD 240109) in the electrodes. The relative amount of β‐LiAl in Alu2 and Alu3 was greater than in Alu1. It should be noted that despite significant amounts of Mg in both Alu2 and Alu3, no Li−Mg alloys are recognizable from the diffractogram. This shows that Li preferentially reacts with Al in the case of single‐phase Al−Mg alloys. The average size of the residual crystals of β‐LiAl in the electrodes retrieved after cycling has been estimated using the Scherrer equation, and it is reported in Figure 3e. This way, the size of β‐LiAl crystals has been estimated to be 31 nm, 39 nm, and 40 nm in Alu1, Alu2, and Alu3, respectively. In agreement with Wang et al.^[29]^ the pulverization occurring in sample Alu1 is responsible for the fast capacity fade observed in Figure 2. On the contrary, Alu3 forms bigger β‐LiAl crystallites, showing better mechanical stability. The accuracy of crystallite size estimation can be limited due to the low amount of β‐LiAl crystals. Figure 4a–f shows the SEM micrographs of Alu1, Alu2, and Alu3 at low magnification. Figure 4a and b shows defects on the surface of the foils due to the lamination process, while Figure 4c shows that the mesh is made of Al wires with a diameter of about 100 μm separated by gaps having approximately the same size. All cycled samples (Figure 4d–f) presented microcracks/pores (indicated by arrows) generated during cycling. Zheng et al. clearly explained that nanopores form during the removal of Li from the β‐LiAl phase and makes this brittle phase and its surroundings even more prone to cracking.^[35]^

**Figure 5:**
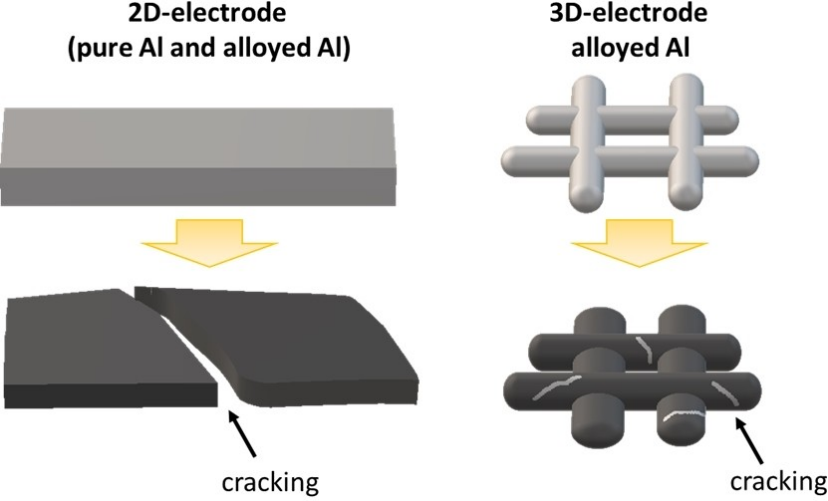
Schematical representation of the influence of alloying and 3D structuring on the electrochemomechanical behavior of Al electrodes.

Figure 5 summarizes our findings: the formation of cracks is inevitable regardless of the morphology of Al (2D or 3D). A 3D Mg‐alloyed aluminum mesh can sustain the mechanical stress better due to the formation of bigger β‐LiAl crystallites (40 nm) with respect to pure Al foil (31 nm). A 3D structure can favor the diffusion of Li, avoiding the irreversible formation of β‐LiAl.

## Conclusions

Two main strategies come into play when it comes to maintaining the high capacity of Al‐based electrodes for many cycles. First, the capacity drop during the initial cycles needs to be minimized: this can be done by facilitating the diffusion of Li from the bulk of the active material to its surface, consequently reducing the irreversible formation of LiAl trapped in the Al matrix. Second, after the capacity stabilizes, it should be retained for as long as possible. This can be done by introducing alloy elements such as Mg into Al. From our results, it can be concluded that, in phase‐pure Al alloys, the benefits of these two approaches are cumulative. For this reason, future researchers who want to obtain Al‐based electrodes with the best possible performance are advised to focus on both the structural and compositional aspects of the electrode. This combined approach is critical to unlocking the best possible stability and capacity for Al‐based electrodes.

## Conflict of Interests

The authors declare no competing financial interest.

1

## Data Availability

The data that support the findings of this study are available from the corresponding author upon reasonable request.
